# Efficacy of first-line treatment in driver gene-negative non-small cell lung cancer with liver metastases: a Bayesian network meta-analysis

**DOI:** 10.3389/fimmu.2026.1791664

**Published:** 2026-07-15

**Authors:** Weiqian Wu, Xiaoyu Guo, Xueqi Dong, Hongyuan Liang, Lingyun Zhang

**Affiliations:** 1 Oncology Department, The First Hospital of China Medical University, Shenyang, China; 2 Department of Radiology, Shengjing Hospital of China Medical University, Shenyang, China

**Keywords:** first-line therapy, immune checkpoint inhibitors, liver metastases, network meta-analysis, non-small cell lung cancer

## Abstract

**Objective:**

This study aimed to evaluate the first-line treatment patterns and prognostic factors associated with survival in patients with driver gene-negative non-small cell lung cancer (NSCLC) and liver metastases, in order to identify the optimal treatment strategy.

**Methods:**

A Bayesian network meta-analysis was performed using R software (version 4.2.3) and RevMan (version 5.4) to systematically compare the efficacy of various first-line treatment regimens, including chemotherapy, immunotherapy, and combination therapy, in patients with driver gene-negative NSCLC with liver metastases.

**Results:**

A total of 20 randomized controlled trials were included. Among patients with driver gene-negative NSCLC and liver metastases: (1) PD-1 inhibitor plus chemotherapy significantly improved progression-free survival (PFS) (HR = 0.572, 95% CI: 0.435–0.754) and overall survival (OS) (HR = 0.681, 95% CI: 0.559–0.830) compared with chemotherapy alone. (2) In the patients with non-squamous NSCLC, PD-1/PD-L1 inhibitor plus chemotherapy resulted in a greater PFS benefit than chemotherapy alone. (3) A similar PFS advantage was observed in patients with squamous NSCLC receiving PD-1 inhibitor plus chemotherapy versus chemotherapy alone (HR = 0.583, 95% CI: 0.386–0.882). (4) Camrelizumab plus chemotherapy (CAM+CT) ranked highest in the network meta-analysis, with the top SUCRA values for both PFS (84.18%) and OS (96.38%).

**Conclusion:**

In treatment-naive driver gene-negative NSCLC with liver metastases: PD-1 inhibitor plus chemotherapy conferred more significant PFS and OS benefits than chemotherapy alone. CAM+CT appeared to rank favorably among the evaluated regimens, particularly in patients with squamous NSCLC and liver metastases, suggesting it may represent a candidate treatment strategy. However, given that these findings were derived from a network meta-analysis based on indirect comparisons, they should be interpreted with caution.

**Systematic Review Registration:**

https://www.crd.york.ac.uk/PROSPERO/view/CRD42025632364, identifier CRD42025632364.

## 1 Introduction

Lung cancer is one of the most common malignancies worldwide. In China, it ranks first in both incidence and mortality. Non-small cell lung cancer (NSCLC) accounts for over 85% of lung cancer cases, with approximately 20% of advanced NSCLC patients developing liver metastases (LMs) (1). Although treatment options for driver gene-negative advanced NSCLC have expanded, including traditional chemotherapy, immunotherapy, and combination strategies, current clinical guidelines do not provide specific recommendations or stratified treatment strategies for patients with LMs. Therefore, further research is needed to identify optimal therapeutic approaches and improve clinical outcomes in this population.

Clinical trials such as ECOG1594 (2), JMDB, and JMIL (3) provided pivotal evidence supporting the use of platinum-based doublet chemotherapy in the treatment of driver gene-negative advanced NSCLC. For more than a decade, chemotherapy remained the primary first-line treatment option for patients with driver gene-negative NSCLC and LMs; however, the survival benefit for this subgroup was markedly limited, with a median overall survival (mOS) of less than 10 months and an objective response rate (ORR) of only 17-22% (4).

In recent years, the development of immunotherapy has led to a paradigm shift in the treatment of advanced NSCLC. Comprehensive treatment strategies centered on programmed cell death protein 1 (PD-1)/programmed cell death ligand 1 (PD-L1) inhibitors are now recommended by both the National Comprehensive Cancer Network (NCCN) and the Chinese Society of Clinical Oncology (CSCO) guidelines for patients with driver gene-negative disease (5). The EMPOWER-Lung 1 study demonstrated that in patients with LMs and PD-L1 expression level ≥ 50%, first-line cemiplimab monotherapy achieved an mOS of 20.4 months, significantly superior to the chemotherapy group (7.6 months), with an ORR of 37.5% (6).

Despite the observed benefits of immunotherapy, patients with LMs exhibit a poorer response compared to the overall population. The presence of LMs has been identified an independent predictor of inferior outcomes with immune checkpoint inhibitor (ICI) monotherapy. A secondary analysis by Topalian et al. (7) from the CA209–003 study, which evaluated nivolumab monotherapy in advanced NSCLC patients, found that baseline LMs were independently associated with reduced 5-year survival rates (OR = 0.31; 95% CI, 0.12–0.83; P = 0.02). Similarly, a retrospective study reported that LMs were a poor prognostic factor for progression-free survival (PFS) in patients with lung adenocarcinoma (LUAD) receiving ICI monotherapy (8). In later-line settings, a pooled analysis of the ATLANTIC and Study 1108 trials by Sridhar et al. (9) further confirmed that patients with LMs receiving durvalumab monotherapy had a shorter median overall survival (mOS) and PFS compared with those without LMs, establishing LMs as an independent adverse prognostic factor.

In contrast, the combination of immunotherapy and chemotherapy has demonstrated significant improvements in both PFS and OS for patients with LMs. The EMPOWER-Lung 3 Part 2 study (6) reported that LM patients treated with cemiplimab combined chemotherapy achieved an ORR of 37.5% and an mOS of 15.5 months, significantly better than the chemotherapy group (8.9 months). Additionally, regimens incorporating immunotherapy with chemotherapy and antiangiogenic therapy have also shown efficacy and potential for LM patients. Results from the IMpower150 study (10) indicated that the ABCP regimen (atezolizumab combined with bevacizumab and chemotherapy) significantly improved both median PFS (mPFS; 8.2 months) and mOS (13.3 months) in patients with LMs.

However, an optimal first-line treatment strategy for driver gene-negative NSCLC with LMs has yet to be established. Moreover, the various therapeutic strategies investigated in clinical trials exhibit disparate efficacy and safety profiles. The absence of a comprehensive and systematic comparison limits clinicians’ ability to make informed decisions and develop individualized treatment strategies for patients with LMs.

Bayesian theory is a statistical approach based on probabilistic inference, allowing integration of prior knowledge with new empirical data to generate more accurate posterior probabilities (11). In medical research, the Bayesian approach is particularly effective for addressing complex data structures and uncertainty, thereby enhancing clinical decision-making. Network meta-analysis (NMA) is an advanced method that facilitates indirect and direct comparisons across multiple treatment strategies. Within a Bayesian framework, NMA can synthesize evidence from diverse studies, providing a more comprehensive evaluation of treatment efficacy. This methodology is especially valuable for evaluating therapeutic options in specific clinical settings where head-to-head trials are lacking.

In light of these considerations, the present study employs a NMA within a Bayesian framework to systematically evaluate the efficacy of various first-line treatment strategies—including chemotherapy, immunotherapy, bevacizumab-based combinations, and others—for patients with driver gene-negative NSCLC and LMs. The aim is to fill the current gap in comprehensive comparative assessments for this specific population and to provide clinicians with an evidence-based tool to guide treatment selection. Additionally, the findings may inform the design of future clinical trials, support the development of high-quality research, and ultimately improve survival outcomes and quality of life for patients with LMs.

## 2 Materials and methods

### 2.1 Systematic literature search

A systematic literature search was conducted following the Preferred Reporting Items for Systematic Reviews and Meta-Analyses (PRISMA) guidelines. The following electronic databases were searched from their inception to June 30, 2025, including PubMed, Embase, and the Cochrane Central Register of Controlled Trials (CENTRAL). Abstracts from major oncology conferences, including the American Society of Clinical Oncology (ASCO), CSCO, World Conference on Lung Cancer (WCLC), and European Society for Medical Oncology (ESMO), were also screened. Additional studies were identified through manual review of reference lists of included articles and relevant reviews. The full search strategy and PRISMA flow diagram are provided in the Supplementary Materials. The study protocol was prospectively registered on International Prospective Register of Systematic Reviews (PROSPERO) under registration number CRD42025632364.

### 2.2 Data extraction and quality assessment

Data extraction was performed independently by two investigators using a predefined, standardized form. The following information was collected: study design, patient characteristics, intervention details, and outcome measures. Hazard ratios (HR) along with corresponding 95% confidence intervals (CI) were extracted for direct analysis. The risk of bias for included randomized controlled trials was independently assessed by two reviewers using the Cochrane Risk of Bias tool (RoB 2) for randomized trials. Any discrepancies encountered during data extraction or quality assessment were resolved through discussion or, when necessary, adjudication by a third reviewer until a consensus was achieved.

### 2.3 Inclusion and exclusion criteria

The inclusion criteria were defined according to the evidence-based PICOS (Population, Intervention, Comparison, Outcomes, Study design) principles:

1. Population: Patients with a pathological or cytological diagnosis of NSCLC, radiologically confirmed LMs, and confirmed wild-type status for epidermal growth factor receptor (EGFR), anaplastic lymphoma kinase (ALK), and c-ros oncogene 1 receptor tyrosine kinase (ROS1).
2. Intervention: Experimental treatments included immune checkpoint inhibitors (ICIs) with or without chemotherapy, which could be combined with antiangiogenic therapy. The control interventions consisted of platinum-based chemotherapy or ICI monotherapy.
3. Outcomes: The primary outcome measure was the HRs for PFS with its 95% CI. The secondary outcome was the HR for OS with its 95% CI.
4. Study design: Randomized controlled trials (RCTs) evaluating the efficacy and safety of first-line treatments in patients with advanced NSCLC.

Studies were excluded based on the following criteria: (1) Phase I or II clinical trials; (2) Studies involving treatment with EGFR tyrosine kinase inhibitors (TKIs) or ALK-TKIs; (3) Systematic reviews, meta-analyses, animal studies, case reports, or retrospective studies; (4) Duplicate publications; (5) Studies lacking the primary outcome data or those for which HRs with 95% CI could not be derived.

### 2.4 Definition of study endpoints

The primary endpoint was PFS, defined as the time from randomization to the first documented disease progression or death from any cause. The secondary endpoint was OS, defined as the time from randomization to death from any cause. HR for all time-to-event endpoints were estimated based on a stratified Cox proportional hazards model.

### 2.5 Statistical analysis

Data organization and the calculation of pooled HR with 95% CI for PFS and OS were performed using RevMan software (version 5.4). Heterogeneity across studies was assessed using the I² statistic and the Q-test. Based on the degree of heterogeneity, either a common-effect (fixed-effect) model or a random-effects model was applied for the pairwise meta-analyses.

A Bayesian NMA was subsequently conducted using R software (version 4.2.3) with the “gemtc” and “rjags” packages. Four Markov chains were established for simulation, with the number of iterations and the burn-in period set at 50,000 and 20,000, respectively.

A network plot was generated to visualize the evidence structure. Consistency or inconsistency models were selected based on the presence of closed loops and the results of node-splitting analyses. Pooled treatment effects from the NMA were reported as HRs with 95% CI. To estimate the ranking probabilities of the different treatment strategies, the surface under the cumulative ranking curve (SUCRA) was calculated, providing both numerical and visual summaries of treatment efficacy. Rankograms were additionally generated to illustrate the overall probability distribution of treatment rankings.

## 3 Results

### 3.1 Literature search and screening results

**Figure 1:**
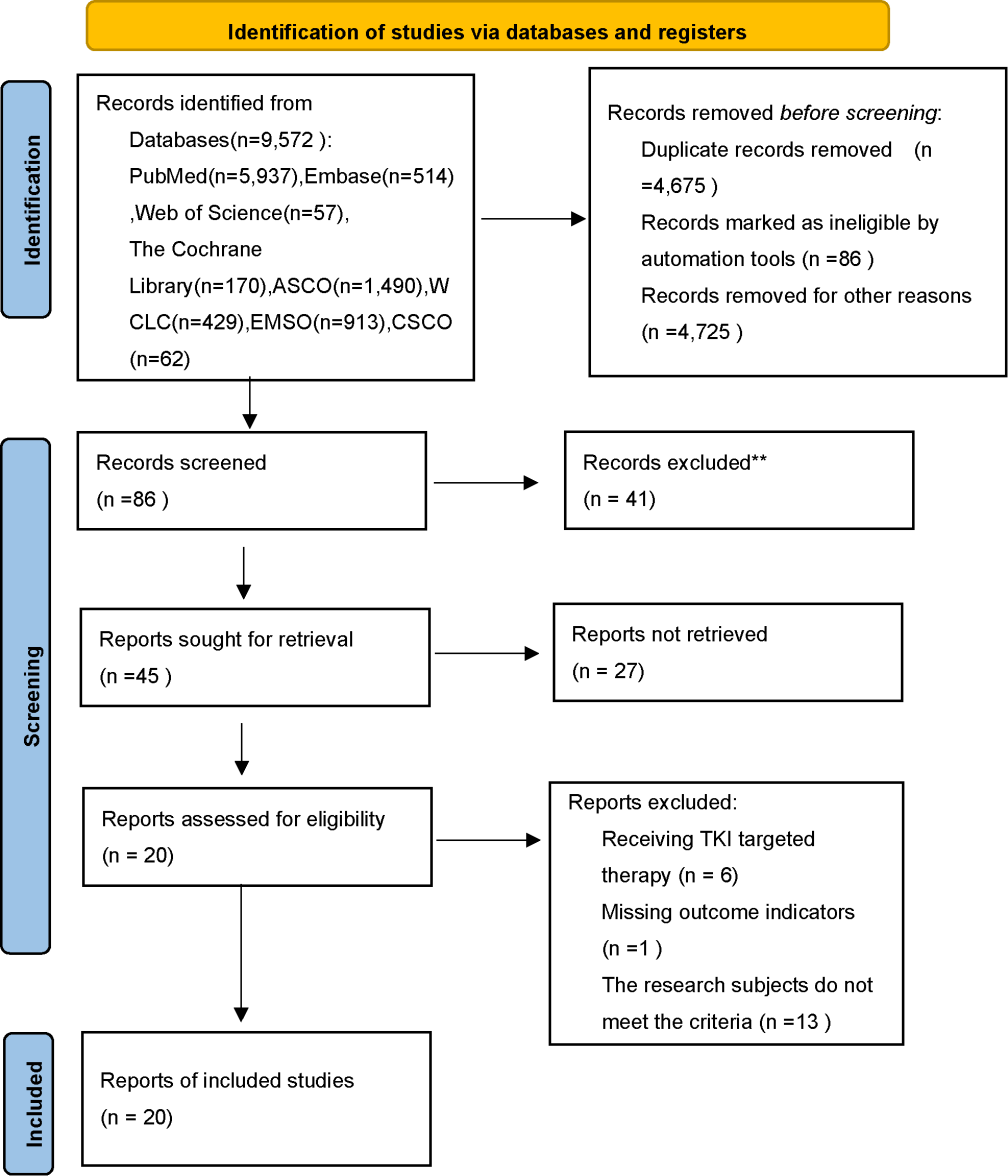
PRISMA flow diagram of literature search and study inclusion process.

The initial systematic search identified 6,678 records, and an additional 2,894 records were retrieved from major oncology conference proceedings. After importing all records into EndNote X8 software and removing duplicates, the titles and abstracts were screened according to the eligibility criteria, resulting in 86 articles selected for full-text review. Following detailed evaluation, 20 RCTs were ultimately included in the quantitative synthesis. These studies collectively enrolled 9,218 patients with advanced driver gene-negative NSCLC, of whom 1,701 had baseline LMs. The study selection process is summarized in the PRISMA flow diagram (**Figure 1**), and the baseline characteristics of the included trials are presented in **Table 1**.

**Table 1:** Basic characteristics of the included studies.

Author	Year	Trial		Number	Experimental arm-LM	Control arm-LM	Treatment	Histological Type	Male, %	HR for PFS	HR for OS	Outcome
Shirish Gadgeel (12)	2020	Keynote189		616	66	49	Pembrolizumab+PC vs. PC+placebo	nSQ NSCLC	363 (58.93)	–	0.62(0.39-0.98)	PFS, OS, safety
A.Baramidze (6)	2023	EMPOWER-Lung 1		565	48	47	Cemiplimab vs. CT	nSQ NSCLC	313 (87.67)	0.43(0.26-0.71)	0.53(0.31-0.89)	PFS, OS, ORR
A.Baramidze (6)	2023	EMPOWER-Lung 3		466	47	23	Cemiplimab+CT vs. placebo+CT	advanced NSCLC	247 (53.00)	0.66(0.37-1.16)	0.65(0.35-1.20)	PFS, OS, ORR
Shirish Gadgeel (12)	2020	IMpower130		679	69	31	Atezolizumab+CT vs. CT	nSQ NSCLC	400(58.91)	0.93 (0.59–1.47)	1.04 (0.63–1.72)	PFS, OS, safety
Alan Sandler (13)	2006	ECOG 4599		850	90	73	Paclitaxel+Carboplatin+Bevacizumab vs. Paclitaxel+Carboplatin	nSQ NSCLC	463 (54.47)	–	0.68 (0.49–0.96)	OS, PFS, ORR, DCR, Safety
Shun Lu (14)	2021	RATIONALE 304		334	20	17	Tislelizumab + CT vs. CT	nSQ NSCLC	247 (74.0)	0.37 (0.15- 0.90)	0.73 (0.51- 1.05)	PFS
Jie Wang (15)	2021	RATIONALE 307-1		241	15	14	Tislelizumab+ PC vs. PC	SQ-NSCLC	218 (90.45)	0.48 (0.19-1.22)	–	PFS
Jie Wang (15)	2021	RATIONALE 307-2		240	15	14	Tislelizumab + nab-PC vs. PC	SQ-NSCLC	223 (92.92)	0.48 (0.19-1.19)	–	PFS
Luis Paz-Ares (16)	2021	CheckMate9LA		719	68	86	Nivolumab+ipilimumab +CT vs. CT	advanced NSCLC	504 (70.10)	–	0.79(0.35-0.89)	OS
Caicun Zhou (17)	2024	ASTRUM-004		537	40	17	Serplulimab+CT vs. CT+ Placebo	SQ-NSCLC	488 (90.88)	1.24(0.51-3.04)	–	PFS, OS, safety, AES
Zhijie Wang (18)	2022	CHOICE-01	465		26	14	Toripalimab+CT vs. CT+Placebo	advanced NSCLC	377 (70.20)	0.64 (0.31-1.36)	1.05 (0.50- 2.35)	PFS, AE
Caicun Zhou (19)	2021	ORIENT-12	357		14	13	Sintilimab+GP vs Placebo-GP	SQ-NSCLC	327 (91.60)	0.61(0.27-1.41)	–	PFS
Shengxiang Ren (20)	2021	CameL-Sq	389		21	19	Camrelizumab+CT vs. Placebo-CT	SQ-NSCLC	359 (92.29)	0.39 (0.19-0.80)	0.35 (0.16-0.77)	PFS
Caicun Zhou (35)	2022	GEMSTONE-302	479		39	18	Sugemalimab+CT vs. Placebo-CT	advanced NSCLC	383 (79.96)	0.54(0.29–1.02)	–	PFS
Makoto Nishio (21)	2020	IMpower132	578		37	36	Atezolizumab+PC vs. PC	nSQ NSCLC	384 (66.40)	0.77(0.47-1.25)	0.96(0.58-1.58)	PFS, OS
S. Sugawara (22)	2021	TASUKI-52	550		19	20	Nivolumab+Bevacizumab+PC vs. PC+Bevacizumab	nSQ NSCLC	411 (74.73)	0.55(0.25-1.23)	–	PFS
Martin Reck (10)	2019	IMpower150-1	800		52	57	Atezolizumab+PC+Bevacizumab vs. PC+Bevacizumab	metastatic NSCLC	479(59.88)	0.41(0.26-0.62)	0.52(0.33-0.82)	PFS, OS, ORR, safety
Martin Reck (10)	2019	IMpower150-2	802		53	57	Atezolizumab+PC vs. PC+Bevacizumab	metastatic NSCLC	480(59.85)	0.81(0.55-1.21)	0.87(0.57-1.32)	PFS, OS, ORR, safety
H. Borghaei (23)	2023	CheckMate 227 part 2	755		86	82	Nivolumab+CT vs. CT	metastatic NSCLC	530 (70.20)	–	0.72(0.50-1.03)	OS, PFS, ORR, safety
Anwen Xiong (24)	2024	HARMONi-2	398		25	28	Ivonescimab vs. Pembrolizumab	advanced NSCLC	333(83.67)	0.47(0.23-0.98)	–	PFS
Baohui Han (25)	2025	CAMPASS	531		30	9	Benmelstobart+Bevacizumab vs. Pembrolizumab	advanced NSCLC	220 (41.43)	0.29(0.11-0.71)	–	PFS, OS, ORR, safety
Yang JC (26)	2023	LEAP-007	623		43	54	Pembrolizumab+Lenvatinib vs. Pembrolizumab	metastatic NSCLC	454(72.87)	0.85(0.53-1.34)	1.05(0.61-1.80)	

### 3.2 Progression-free survival analysis

**Figure 2:**
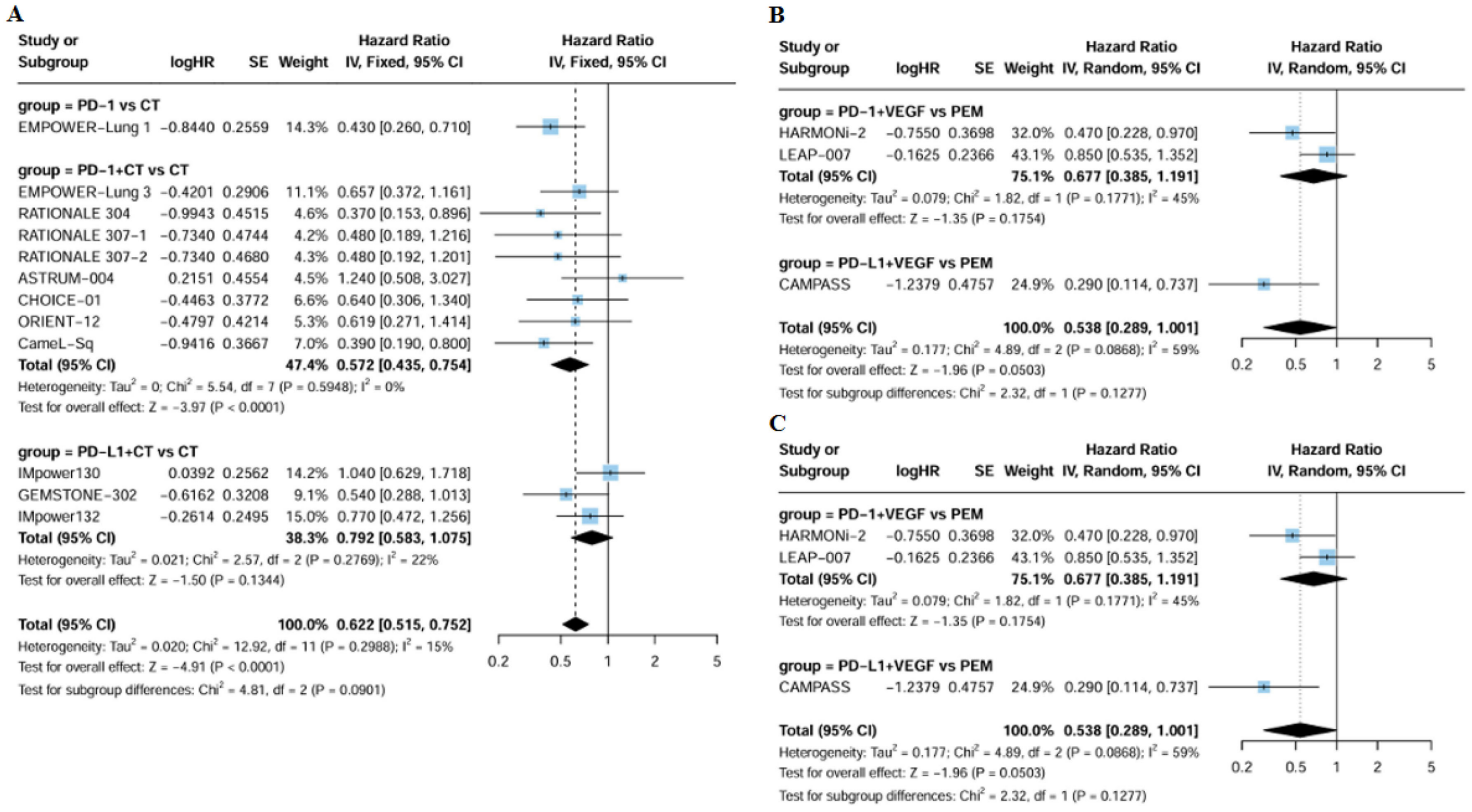
Forest plots from a standard meta-analysis evaluating the impact of different treatment strategies on PFS in patients with LMs, using different control groups: **(A)** Chemotherapy Alone, **(B)** Pembrolizumab Alone, and **(C)** Chemotherapy Combined with Bevacizumab. PD-1, programmed cell death-1; PD-L1, programmed cell death ligand 1; CT, chemotherapy; Beva, Bevacizumab; PEM, Pembrolizumab; VEGF, vascular endothelial growth factor inhibitor.

A total of 16 RCTs reporting PFS data were included in the analysis. Subgroup analyses based on treatment strategy yielded the following findings:

Compared with chemotherapy alone (CT), the pooled analysis revealed low heterogeneity among the studies (I² = 15%, P = 0.30), supporting the use of a fixed-effect model. PD-1 inhibitor plus chemotherapy (PD-1+CT) significantly prolonged PFS (HR = 0.57, 95% CI: 0.44–0.75; P < 0.001), whereas PD-L1 inhibitor combined with chemotherapy (PD-L1+CT) did not demonstrate a significant PFS benefit compared to CT alone (P > 0.05; **Figure 2**). In patients with high PD-L1 expression (tumor proportion score [TPS] ≥ 50%), PD-1 inhibitor monotherapy significantly reduced the risk of disease progression by 57% compared to chemotherapy (HR = 0.43, 95% CI: 0.31–0.60; P < 0.001, **Figure 2A**).

Among patients with LMs, combinations of PD-1/PD-L1 inhibitors with anti-VEGF agents showed a trend toward improved PFS compared with pembrolizumab monotherapy (**Figure 2B**). Furthermore, regimens combining PD-1/PD-L1 inhibitors with either chemotherapy or bevacizumab showed significantly significant PFS benefits compared with CT+ bevacizumab (Beva) (HR = 0.58, 95% CI: 0.36-0.93; P = 0.02; **Figure 2C**).

#### 3.2.1 Non-squamous non-small cell lung cancer

**Figure 3:**
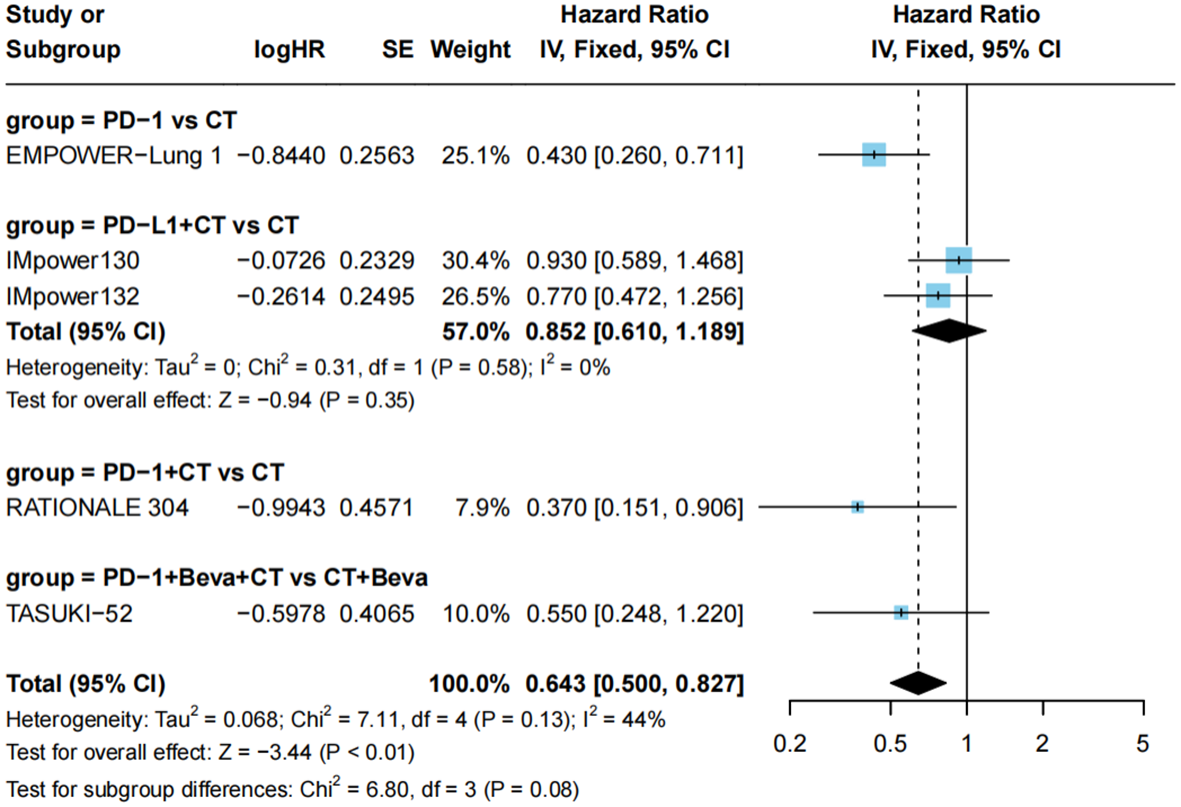
Forest plots from a standard meta-analysis evaluating the impact of different treatment strategies on PFS in nSQ-NSCLC patients with LMs.

Five RCTs (n=344) reported PFS outcomes for the nSQ-NSCLC subgroup. Immunotherapy-based regimens significantly reduced the risk of disease progression (HR = 0.643, 95% CI: 0.500–0.827; P < 0.001), with mild heterogeneity observed among these studies (I² = 44%, P = 0.13). Compared with CT alone, PD-1+CT significantly prolonged PFS (HR = 0.370, 95% CI: 0.151–0.906; P < 0.001). In contrast, neither PD-L1+CT nor PD-1+Beva+CT demonstrated a statistically significant PFS improvement compared to CT. Furthermore, in patients with high PD-L1 expression, PD-1 inhibitor monotherapy was associated with a significant PFS improvement compared to CT (HR = 0.430, 95%CI: 0.260–0.711; P<0.001, **Figure 3**).

#### 3.2.2 Squamous non-small cell lung cancer

**Figure 4:**
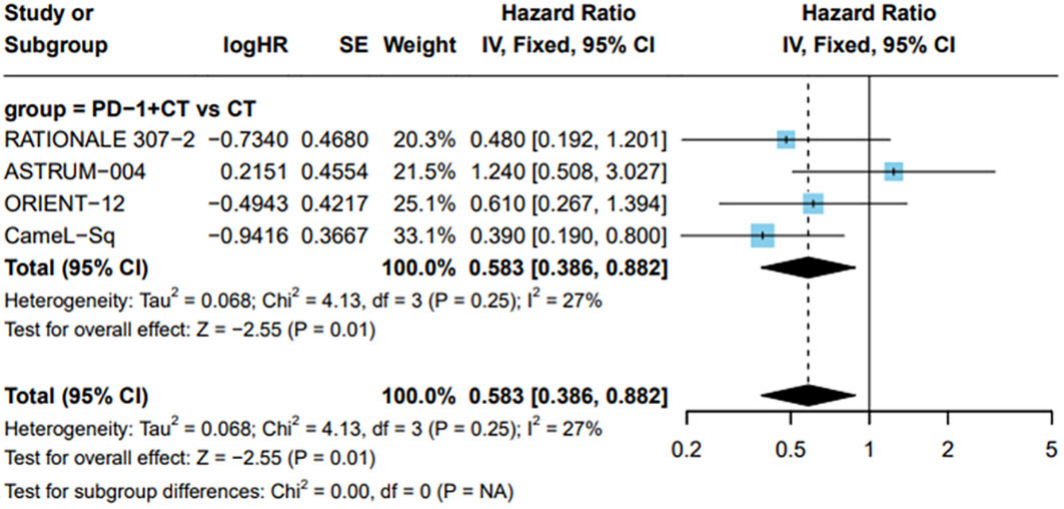
Forest plots from a standard meta-analysis evaluating the impact of different treatment strategies on PFS in squamous patients with LMs.

Four RCTs (n=153) evaluated patients with SQ-NSCLC and liver metastases. All included studies utilized a PD-1+CT regimen. The pooled analysis revealed mild heterogeneity (I² = 27%, P = 0.25) among the studies. Compared with CT, PD-1+CT significantly reduced the risk of disease progression by 41.7% (HR = 0.583, 95% CI: 0.386–0.882; P = 0.011; **Figure 4**).Furthermore, the PFS benefit derived from PD-1+CT treatment was significantly greater in patients with nSQ-NSCLC and LMs compared to those with the SQ-NSCLC and LMs (P for interaction < 0.05).

### 3.3 Overall survival analysis

**Figure 5:**
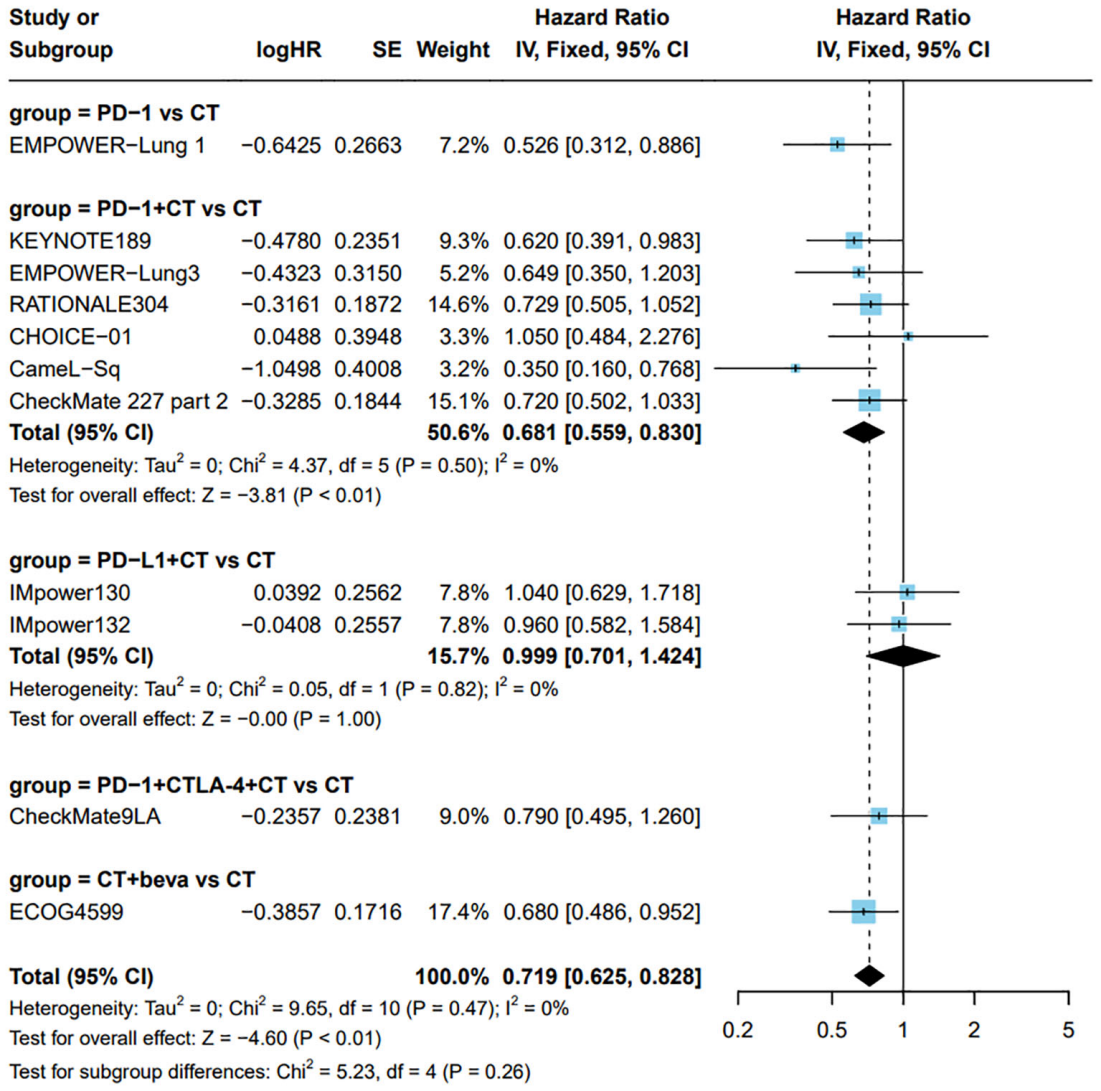
Forest plots from a standard meta-analysis evaluating the impact of different treatment strategies on OS in patients with LMs.

Direct pairwise comparisons were performed based on OS data from 11 RCTs. No significant heterogeneity was observed (I² = 0%, P = 0.26, **Figure 5**), supporting the use of a fixed-effect model. Compared with CT, PD-1+CT significantly reduced the risk of death by 31.9% in patients with LMs (HR = 0.681). Similarly, CT+Beva was produced a similar mortality reduction (32%, HR = 0.680). PD-1 inhibitor combined with CTLA-4 inhibitor and chemotherapy (PD-1+CTLA-4+CT) reduced the risk of death by 21% (HR = 0.790). In contrast, the comparison between PD-L1+CT and CT alone showed no statistically significant difference (HR = 0.999). Among patients with high PD-L1 expression, PD-1 inhibitor therapy provided a more pronounced OS benefit compared with chemotherapy (HR = 0.526). In this study, with the exception of the CameL-Sq trial, none of the remaining RCTs involving patients with SQ-NSCLC and LMs reported OS data. The limited availability of single-study outcomes precluded meaningful analysis.

**Figure 6:**
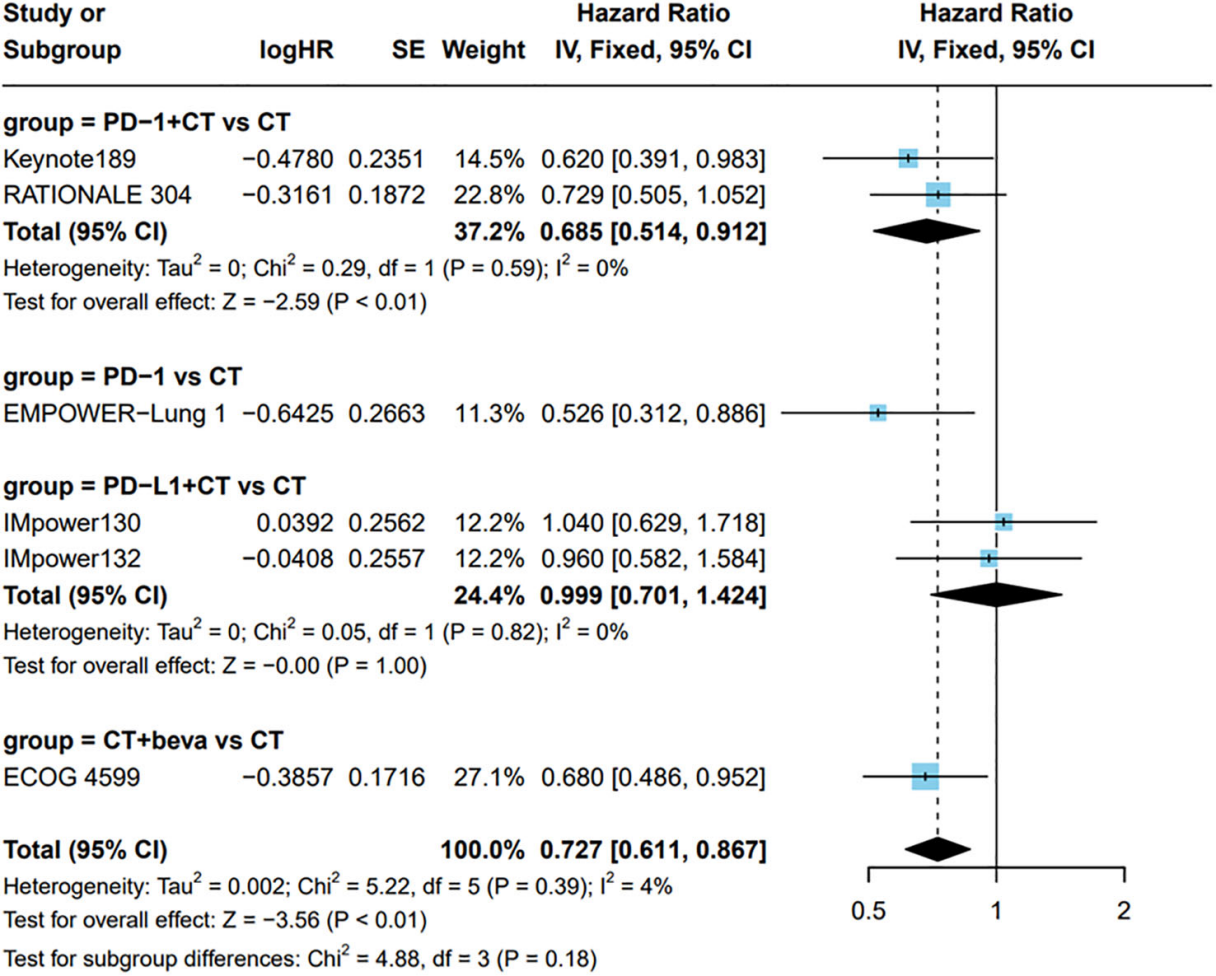
Forest plots from a standard meta-analysis evaluating the impact of different treatment strategies on PFS in nSQ-NSCLC patients with LMs.

Six RCTs reported OS data for patients with nSQ-NSCLC (**Figure 6**). The pooled analysis showed that immunotherapy-based regimens significantly improved OS compared with chemotherapy (HR = 0.727, 95% CI: 0.611–0.867; P < 0.01). Heterogeneity was low (I² = 4%, P = 0.39), supporting the use of a fixed-effect model. Compared to the CT group, both the PD-1+CT group (HR = 0.685) and the CT+Beva group (HR = 0.680) provided greater reductions in mortality risk. Among patients with high PD-L1 expression, PD-1 inhibitor monotherapy reduced the risk of death by 47.4% (HR = 0.526). In contrast, PD-L1+CT group did not show a statistically significant OS benefit compared to CT (P = 1.00).

### 3.4 Network meta-analysis

**Figure 7:**
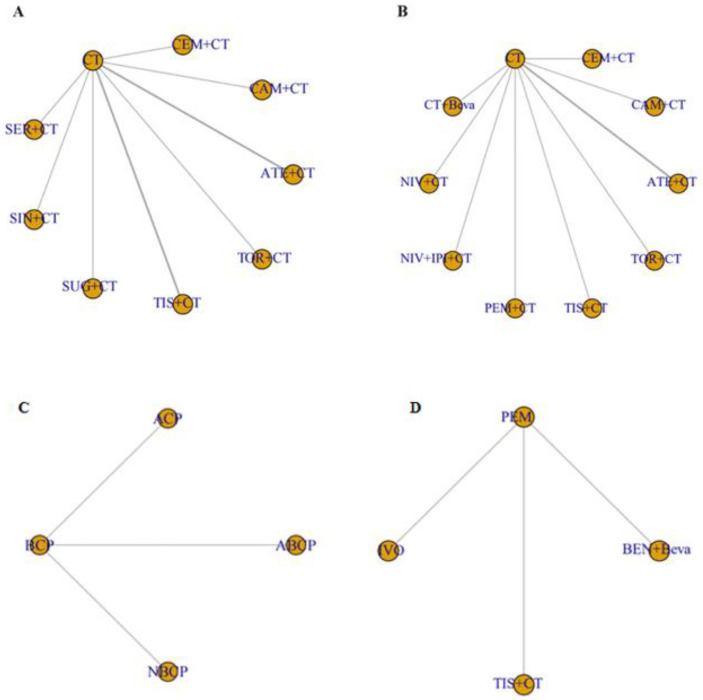
Network plots for **(A)** PFS vs CT, **(B)** OS vs CT, **(C)** PFS vs BCP, and **(D)** PFS vs PEM. CT, chemotherapy; ATE, atezolizumab; CAM, camrelizumab; CEM, cemiplimab; SER, serplulimab; SIN, sintilimab; SUG, sugemalimab; TIS, tislelizumab; TOR, toripalimab; PEM, Pembrolizumab; NIV, Nivolumab; Beva, bevacizumab; IPI, ipilimumab; IVO, Ivonescimab; BEN, Benmelstobart; ABCP, atezolizumab plus bevacizumab plus carboplatin plus paclitaxe; BCP, bevacizumab plus carboplatin plus paclitaxel; NBCP, nivolumab plus bevacizumab plus carboplatin plus paclitaxel; ACP, atezolizumab plus carboplatin plus paclitaxel.

A Bayesian NMA was performed to compare and rank the relative efficacy of various first-line treatment strategies for patients with driver gene-negative NSCLC and LMs (**Figure 7**).

#### 3.4.1 Progression-free survival

**Figure 8:**
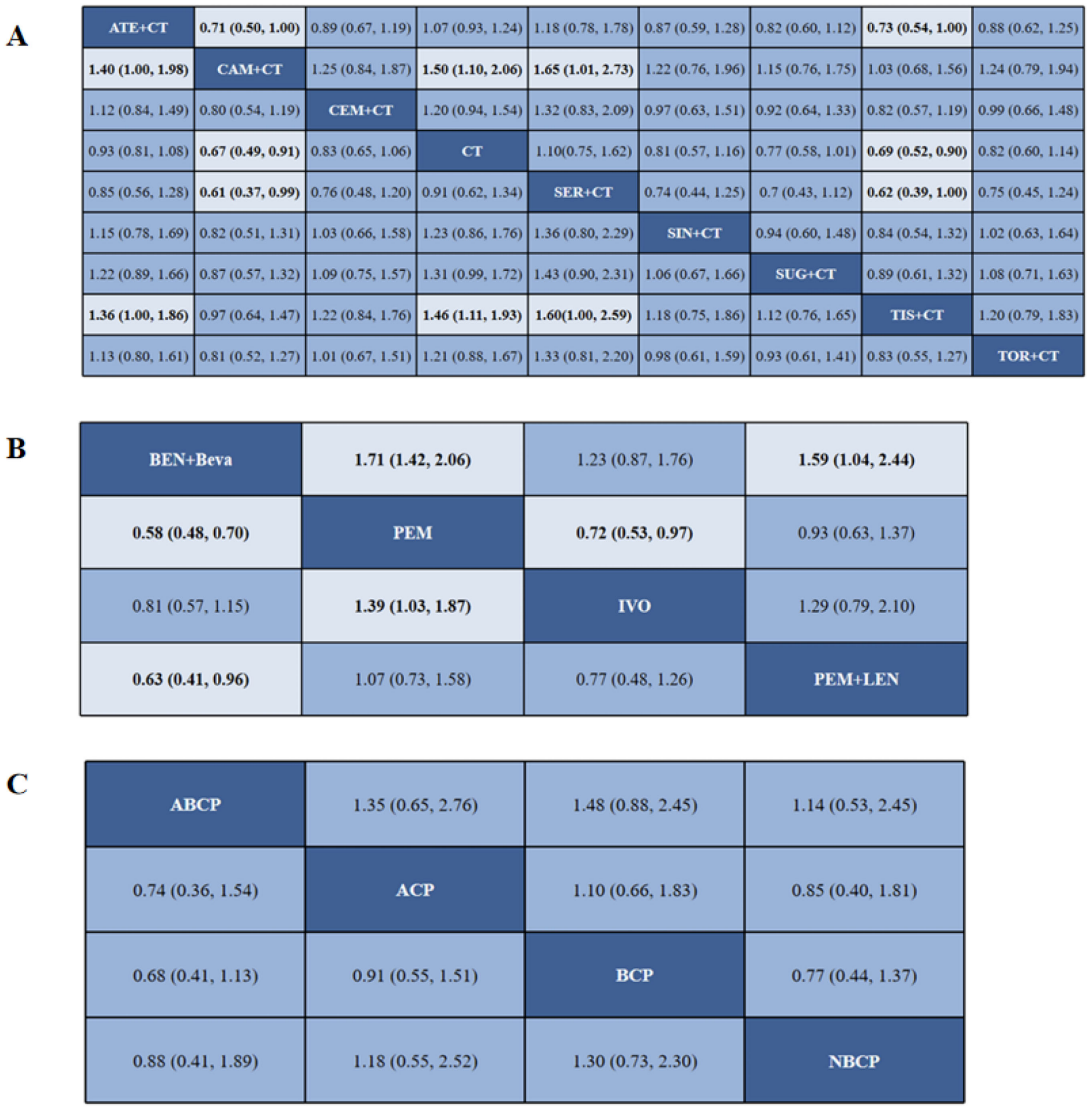
League table for PFS-related outcome measures with **(A)** CT, **(B)** PEM, and **(C)** BCP as control.

The league table of pairwise comparisons is presented in **Figure 8A**. Compared to CT, CAM+CT significantly improved PFS (HR = 0.67, 95% CI:0.49-0.91). Tislelizumab combined with chemotherapy (TIS+CT) also demonstrated a significant PFS benefit over CT (HR = 0.69, 95% CI: 0.52–0.90). TIS+CT demonstrated greater PFS benefit than SER+CT and ATE+CT, while CAM+CT was superior to SER+CT in patients with LMs. No other pairwise comparisons showed statistically significant differences.

**Figure 9:**
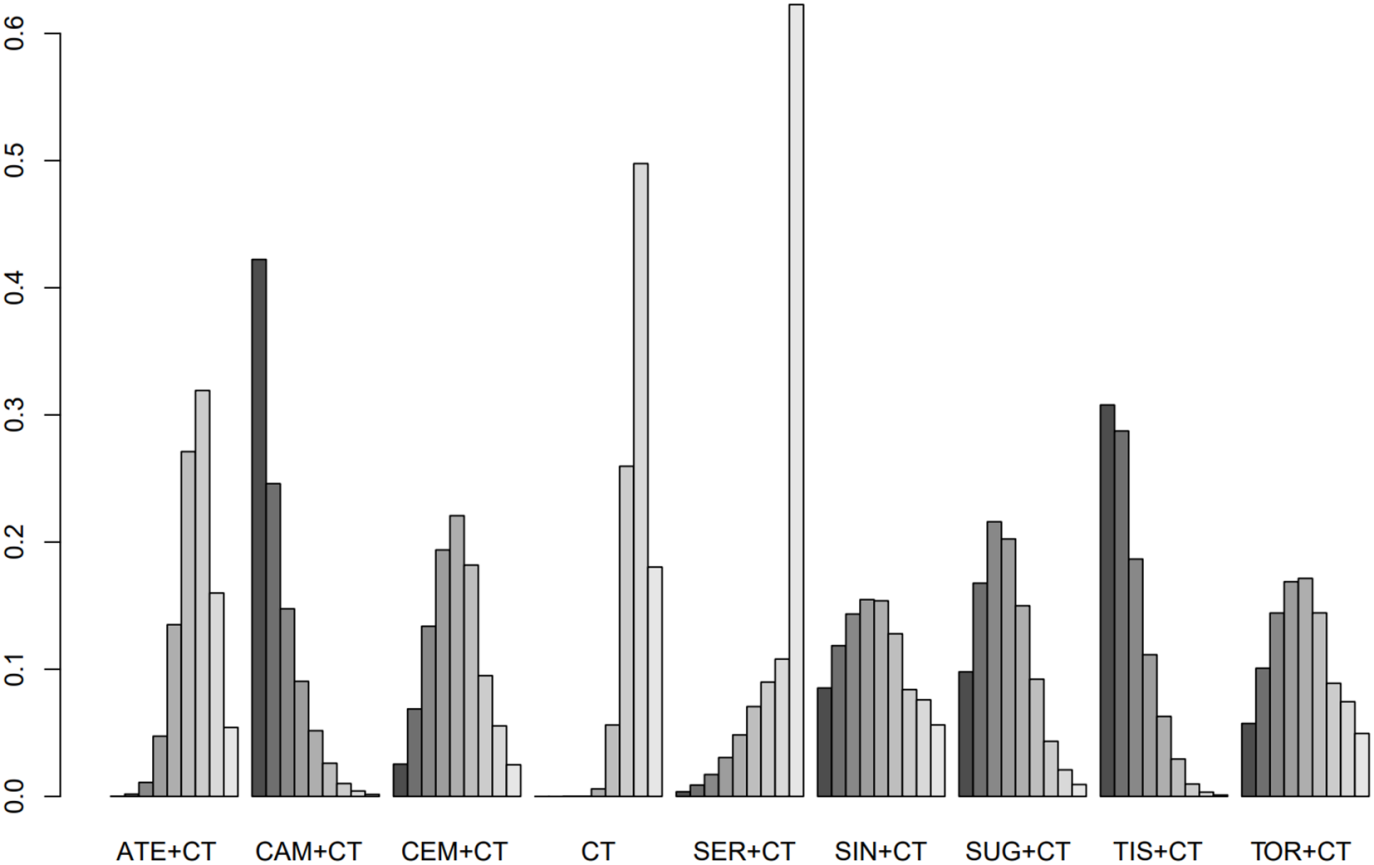
Histogram of ranking distributions for different treatment regimens based on PFS-related outcome measures. For each treatment regimen on the horizontal axis, the leftmost bar corresponds to the probability of achieving rank 1 (first-place ranking), with subsequent bars sequentially denoting the probabilities of rank 2, rank 3 and onward; higher values indicate a more favorable ranking for PFS. The horizontal axis represents different treatment regimens, and the vertical axis represents the ranking metric. In terms of first-rank probability, CAM+CT has the highest value of 42.23%, followed by TIS+CT (30.79%), SUG+CT (9.80%), SIN+CT (8.53%), TOR+CT (5.74%), CEM+CT (2.54%), SER+CT (0.37%), ATE+CT (0.02%), and CT (0.00%).

The hierarchy of treatment efficacy based on Surface Under the Cumulative Ranking Curve (SUCRA) values, from highest to lowest, was as follows: CAM+CT (84.18%), TIS+CT (81.50%), sugemalimab combined with chemotherapy (SUG+CT; 65.61%), sintilimab combined with chemotherapy (SIN+CT; 55.15%), toripalimab combined with chemotherapy (TOR+CT; 52.94%), cemiplimab combined with chemotherapy (CEM+CT; 51.77%), atezolizumab combined with chemotherapy (ATE+CT; 30.88%), CT (15.17%), and SER+CT (12.81%). Higher SUCRA values indicate a greater probability of being the most effective regimen. Although NMA included mixed histologies, the trial for CAM+CT was conducted predominantly in SQ-NSCLC; therefore, its top ranking for PFS is most directly applicable to the squamous subgroup (**Figure 9**).

Compared to PEM, both benmelstobart plus bevacizumab (BEN+Beva) and IVO demonstrated superior PFS benefits. Furthermore, BEN+Beva also significantly prolonged PFS compared to pembrolizumab plus Lenvatinib (PEM+LEN). SUCRA rankings further supported these findings: BEN+Beva (84.06 percent) ranked highest, followed by IVO (62.35%), PEN+LEN (33.50%), and PEM (20.09%). This order is consistent with the results presented in **Figure 8B**.

When using BCP as the reference in the league table, no statistically significant differences were observed between ABCP, ACP, and NBCP (**Figure 8C**).

#### 3.4.2 Overall survival

**Figure 10:**
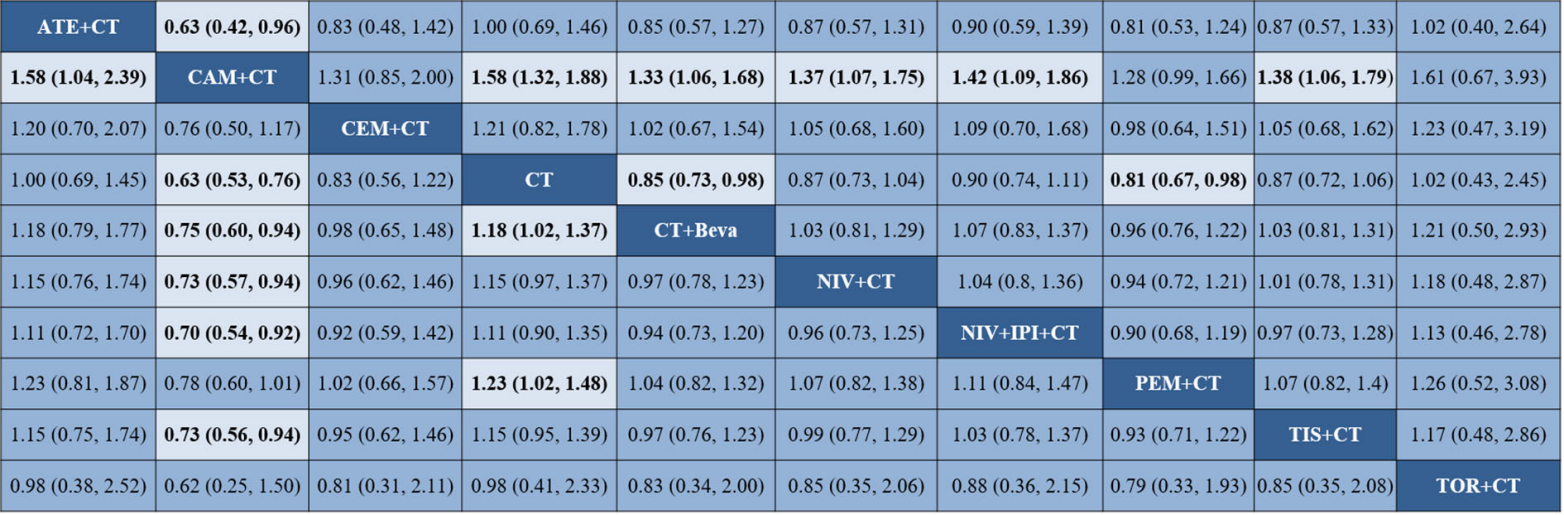
League table for OS-related outcome measures with CT as control.

This study included 9 RCTs and performed a network meta-analysis of the OS across 10 treatment strategies. The league table (**Figure 10**) indicated that, compared with CT, the following regimens significantly improved OS: CAM+CT (HR = 0.63, 95% CI: 0.53-0.76); PEM+CT (HR = 0.81, 95% CI: 0.67-0.98); CT+Beva (HR = 0.85, 95% CI: 0.73-0.98). Immunotherapy plus chemotherapy regimens reduced the risk of death by at least 19% compared to CT alone, and demonstrated a superior OS benefit compared to CT+Beva. Notably, CAM+CT reduced the risk of death by 25% compared to CT+Beva (HR = 0.75, 95% CI: 0.60-0.94). Among all evaluated strategies, demonstrated the greatest survival advantage in patients with LMs. The SUCRA rankings for OS, from highest to lowest, were as follows: CAM+CT (96.38%), PEM+CT (65.10%), CEM+CT (57.50%), CT+Beva (57.40%), NIV+CT (51.16%), TIS+CT (49.81%), NIV+IPI+CT (42.36%), TOR+CT (35.66%), ATE+CT (27.81%), and CT (16.83%). These results suggest that CAM+CT is most likely the optimal therapeutic strategy for improving OS in patients with LMs, based on evidence primarily derived from a SQ-NSCLC population.

## 4 Discussion

This systematic review evaluated first-line treatments for driver gene-negative NSCLC patients with LMs. The findings demonstrate that ICIs, particularly when combined with chemotherapy, significantly improved both PFS and OS compared to chemotherapy alone, underscoring treatment strategy as an important prognostic determinant. These findings align with existing evidence, including a real-world study reporting prolonged survival in LMs patients treated with ICIs (ORR: 29.5%; DCR: 72.1%; mPFS: 6.4 months; mOS: 15.2 months) (27). Moreover, multiple prospective trials have consistently demonstrated the superior efficacy of ICI-chemotherapy combinations over CT alone (28, 29).

This study revealed that nSQ-NSCLC patients derived greater PFS benefits from PD-1 inhibitor plus CT than those with SQ-NSCLC, indicating histological subtype may serve as a potential prognostic factor for disease progression in LMs. The higher propensity for nSQ-NSCLC to develop LMs (reported incidence in advanced NSCLC: 13.4%-22%) may partly account for this observation (1, 30, 31). In contrast, SQ-NSCLC patients exhibit poorer outcomes following ICI-based therapy, which is likely attributable to distinct genomic features, cellular composition, and tumor immune microenvironment (TIME) features. Compared to lung adenocarcinoma (LUAD), lung squamous cell carcinoma (LUSC) shows higher levels of neutrophil infiltration, which neutrophils being recruited and modulated through SPP1 and JAG–NOTCH signaling, thereby contributing to an immunosuppressive TIME (32, 33). High neutrophil levels are linked to ICI resistance (34), which is consistent with observed lower response rates in LUSC relative to LUAD (35–37).

This study found that PD-1 inhibitors combined with chemotherapy provided superior PFS and OS benefits over PD-L1-based combinations. Although direct head-to-head trials are not available, indirect evidence supports this finding. A cross-trial analysis by Duan et al. (38) involving 19 RCTs across solid tumors demonstrated that PD-1 inhibitors were associated with improved OS (HR = 0.75) and PFS (HR = 0.73). This advantage may be explained by mechanistic differences between the two classes of agents. PD-1 inhibitors block both PD-L1 and PD-L2 binding, enabling broader immune activation and potentially reducing ligand compensation-mediated resistance (39). This broader blockade may be particularly beneficial in tumors with low PD-L1 expression or high spatial heterogeneity. Additionally, by directly targeting the PD-1 receptor on T cells, PD-1 inhibitors more effectively reverse downstream inhibitory signaling mediated through ITIM and ITSM motifs (40), whereas PD-L1 inhibitors primarily disrupt ligand-receptor interaction with influencing intracellular signaling pathways.

In this Network meta-analysis, CAM+CT appeared to rank favorably among the evaluated regimens. However, these findings should be considered exploratory rather than definitive. The observed ranking differences may be influenced by factors beyond molecular characteristics, including variations in patient populations (e.g., demographic features, disease stage, histological subtypes, and baseline liver metastasis burden), trial design, and the time periods during which the included studies were conducted. Although preclinical studies have suggested certain mechanistic advantages of CAM (41) (42), whether these differences translate into clinically meaningful superiority over other PD−1 inhibitors remains speculative and requires further validation. Therefore, the following interpretation should be regarded as hypothesis-generating rather than conclusive: these preclinical observations may underlie the demonstrated clinical efficacy of CAM in advanced NSCLC, both in combination with chemotherapy and antiangiogenic agents, and support its approvals across multiple tumor types (43, 44). Future head−to−head randomized controlled trials directly comparing CAM−based regimens with other PD−1/PD−L1 inhibitors in well−stratified patient populations (especially those with liver metastases) are warranted to determine whether the observed ranking differences reflect true therapeutic superiority or are influenced by confounding factors.

Notably, this study found that combination therapy with anti-VEGF agents not only significantly prolonged PFS but also improved OS benefits in patients with advanced NSCLC and LMs. Bevacizumab, one of the most widely used anti-angiogenic agents, selectively targets VEGF and alleviates hypoxia in the TIME, and has demonstrated significant efficacy in the treatment of NSCLC with LMs. Recent studies have shown that when Beva + PD-1 inhibitors enhances CD8+ T cell infiltration and cytotoxic activity, induces sustained anti-tumor effects, and thereby improves the efficacy of ICIs and extends patient survival (45).

Several limitations should be acknowledged. Despite a comprehensive literature search, the included RCTs showed inherent heterogeneity. Differences in trial design and reporting over time may introduce potential bias. Moreover, as all RCTs focused on broad advanced NSCLC populations rather than LMs-specific cohorts, safety comparisons for patients with LMs were not feasible, highlighting the importance of individualized risk assessment in practice. Furthermore, our study primarily focused on first-line systemic treatment regimens, including chemotherapy and PD-1/PD-L1 inhibitor-based therapies, for patients with NSCLC and liver metastases. The potential role of local therapy, such as radiotherapy or hepatic arterial interventional therapy, in combination with first-line systemic treatment remains unclear, as the available randomized evidence is insufficient to evaluate this strategy. Therefore, the therapeutic effects of such combinations could not be assessed in this network meta-analysis. Further prospective studies are warranted to determine whether adding local therapy to first-line systemic treatment provides additional clinical benefits in this specific patient population.

## 5 Conclusion

This NMA offers three key contributions to the treatment landscape of LMs in advanced NSCLC. First, by synthesizing direct and indirect evidence from 18 treatment strategies—including immunotherapy combinations, antiangiogenic regimens, and chemotherapy—we developed the first efficacy ranking specifically for NSCLC with LMs. Second, using Bayesian network meta-analysis and SUCRA, we probabilistically ranked treatment strategies, addressing the absence of head-to-head trials and supporting clinical decision-making for this high-risk population. Camrelizumab plus chemotherapy emerged as the most favorable regimen.

## Data Availability

The original contributions presented in the study are included in the article/supplementary materials. Further inquiries can be directed to the corresponding author.
